# Real-World Adherence and Drug Survival of Biologics among Patients with Ankylosing Spondylitis

**DOI:** 10.3390/jcm13154480

**Published:** 2024-07-31

**Authors:** Vered Rosenberg, Howard Amital, Gabriel Chodick, Freddy Faccin, Abdulla Watad, Dennis McGonagle, Omer Gendelman

**Affiliations:** 1 Maccabi Institute for Research and Innovation, Maccabi Healthcare Services, Tel Aviv 6801296, Israel; rozenb_ve@mac.org.il (V.R.); chodick@tauex.tau.ac.il (G.C.); 2 Faculty of Medicine, Tel Aviv University, Tel Aviv 6997801, Israel; howard.amital@sheba.health.gov.il (H.A.); watad.abdulla@gmail.com (A.W.); 3 Internal Medicine B, Sheba Medical Center, Tel-Hashomer, Ramat Gan 5262000, Israel; 4 AbbVie Inc., North Chicago, IL 60064, USA; freddy.faccin@abbvie.com; 5 Leeds Institute of Rheumatic and Musculoskeletal Medicine, University of Leeds, Leeds LS9 7JT, UK; d.g.mcgonagle@leeds.ac.uk; 6 Leeds Musculoskeletal Biomedical Research Centre, Chapel Allerton Hospital, Leeds LS7 4SA, UK

**Keywords:** ankylosing spondylitis, adherence, drug survival, biological therapy, real world

## Abstract

**Objectives**: The objective of this study was to evaluate the real-world drug survival, adherence, and discontinuation risk of biologics disease-modifying anti-rheumatic drugs (bDMARDs) among patients with ankylosing spondylitis (AS). **Methods**: This was a retrospective study using a computerized database. Biologic-naïve and biologic-experienced AS patients who initiated treatment with bDMARDs (tumor necrosis factor alpha inhibitors {TNF-αis} or interleukin-17 inhibitor {IL-17i}) during 2015–2018 were included. Adherence was assessed using the proportion of days covered (PDC) method. Drug survival was analyzed using Kaplan–Meier estimates. Risk of discontinuation was estimated by the Cox proportional hazard model. **Results**: We identified 343 eligible patients utilizing 481 lines of therapy. The mean age was 44.6 years (SD ± 13.4), 57.7% were males, and 69.7% were biologic-naïve at baseline. The proportion of highly adherent patients (PDC ≥ 0.8) in the biologic-naïve group was 63.5% for golimumab, 69.2% for etanercept, and 71.6% for adalimumab (*p* > 0.9). Among the biologic-experienced group, secukinumab had the highest proportion of adherent patients (75.7%) and etanercept the lowest (50.0%) reaching statistical difference (*p* < 0.001). The Kaplan–Meier analysis did not show a significant difference in drug survival in either the biologic-naïve or the biologic-experienced groups (*p* = 0.85). Multivariable analysis demonstrated a similar risk for discontinuation for etanercept, golimumab, and secukinumab compared with adalimumab, regardless of biologic-experience status. **Conclusions**: Adherence, drug survival, and risk for discontinuation were similar for all TNF-αis and the IL-17i SEC, regardless of biologic-experience status. As drug survival is an indirect measure of drug efficacy, *n,* in real-world settings, we believe caregivers can integrate these results into treatment considerations.

## 1. Introduction

Ankylosing spondylitis (AS) is a chronic, multi-systemic inflammatory disorder which is a member of the spondyloarthropathy group [1]. It primarily affects the sacroiliac joints and the axial skeleton, manifesting clinically as inflammatory back pain and stiffness [2]. Peripheral arthritis, along with enthesitis and dactylitis, is found in about 30% of patients [3,4]. In addition, extra-articular manifestations, most commonly acute anterior uveitis, psoriasis, and inflammatory bowel disease, [5] can occur, embroiling the patient’s condition and treatment decisions [6]. In some cases, chronic axial inflammation can result in progressive structural damage, leading to impaired spinal mobility and postural abnormalities [7,8]. Such irreversible damage is associated with impaired quality of life [7] and social and economic burden [8]. Hence, treatment goals need to be integrative, abrogating inflammation, reducing overall disease activity, and halting structural damage in order to preserve normal function and social participation [9].

Currently, following NSAID treatment failure, the pharmacological armamentarium includes biological disease-modifying anti-rheumatic drugs (bDMARDs), tumor necrosis factor alpha (TNF-α) inhibitors, anti-interleukin (IL)-17A antibodies, and the latest addition in the form of targeted synthetic (ts) DMARDs, Janus kinase (JAK) inhibitors [10]. These therapies have revolutionized [11] care and prognosis for AS patients [12], bringing significant clinical relief and halting disease progression and spinal damage [13].

By and large, the efficacy and safety of the different treatment options seem to be similar [14]. However, data regarding these agents are obtained from placebo-controlled trails, significantly lacking comparative head-to-head studies [9].

Several studies examined biologic drug survival among AS patients [15,16,17,18,19], yet these studies are heterogeneous and differ by follow-up period, the variety of drugs included, patients’ characteristics, and local healthcare systems’ regulations and financing. Therefore, we aimed to compare the real-world adherence and drug survival of different bDMARDs in adult AS patients.

## 2. Methods

### 2.1. Study Design and Data Source

This retrospective cohort study was conducted using the computerized databases of Maccabi Healthcare Services (MHS). MHS is the second largest state-mandated health provider operating in Israel, providing healthcare services for over 2.6 million members (25% of the Israeli population). The MHS databases integrate data from the MHS central laboratory, medication prescriptions, and purchases throughout the MHS pharmacy network, primary care, expert consultations, hospitalizations, procedures, and socio-demographic data. Physician diagnoses are coded using the International Classification of Disease, 9th Edition (ICD-9-CM), codes, as well as internal MHS codes for sub-classification.

This study was conducted in accordance with the protocol, applicable regulations, and guidelines governing clinical study conduct and the ethical principles that have their origin in the Declaration of Helsinki. The MHS Institutional Review Board (IRB) approved the study protocol and related documents. MHS’s IRB waived the requirement to obtain any informed consent for this secondary analysis of existing data (approval number 0108-18-BBL, 18 December 2018).

### 2.2. Study Population and Follow-Up

According to the Israeli regulatory guidelines, AS patients are eligible for bDMARDs or tsDMARDs if they fulfill prerequisite requirements. Prior to procurement, patients are required to receive an authorization from the MHS drugs authorization center, confirming they comply with the guidelines.

The study population included patients who first purchased ≥ 1 of the following drugs between 1 January 2015 and 31 December 2017 for the indication of AS: TNF-α inhibitors (adalimumab {ADA}, infliximab {IFX}, golimumab {GLM}, etanercept {ETN}, certolizumab pegol {CTZ}); IL-17 inhibitor (secukinumab {SEC}). The additional IL-17 inhibitor was not available for MHS patients during the study follow-up period; neither were the JAK inhibitors tofatacitinib and upadacitinib. All patients were prescribed with these drugs for the indication of AS only, according to the MHS drug authorization center. The first purchase was defined as the index date for the study. Included patients were adults (age ≥ 18 years) and MHS members for ≥12 months before and after the index date. During the study follow-up period, all the aforementioned drugs were available as first-line treatment after utilizing ≥ 2 different non-steroidal anti-inflammatory drugs (NSAIDs).

Patients were followed until the earliest of the following dates: death, leaving the MHS, or the end of the follow-up period (31 December 2018).

### 2.3. Study Variables

We included biologic-naïve patients (those with no previous purchase of any of the drugs included in the study before 1 January 2015), as well as biologic-experienced patients (those treated with any of the drugs included in the study before 1 January 2015, or who switched and started treatment with any of the other drugs included in the study, between 1 January 2015 and 31 December 2017).

Initiation of a new line of therapy was defined by the first purchase of the drug. For all patients, we followed all lines of therapy used during the study follow-up period and numbered them. For those defined as biologic-experienced when entering the study, we numbered the lines of therapy used during the study follow-up period, using data on bDMARDs dispensed up to 10 years before entering the study.

Adherence in the first 12 months was evaluated by using the proportion of days covered (PDC) method. PDC reflects the number of days covered by the dispensed drug divided by the total follow-up time for a specific line of therapy. PDC was categorized as follows: non-adherent (PDC < 40%), moderately adherent (40% ≤ PDC < 80%), or highly adherent (PDC ≥ 80%) [20,21,22,23]. For the sake of adherence in the first 12 months, patients were followed from the treatment initiation until the earliest of the following: switching, end of study follow-up, and 12 months after treatment initiation. Only lines of therapy in which patients had at least three months’ follow-up were included.

Drug survival was measured from the initiation of treatment (i.e., the index date) until treatment discontinuation, defined as the first gap of 120 days or more after the last supply date. Patients who discontinued their current drug were further classified as follows: switching (starting a new treatment, with any of the drugs included in the study) or stopping (≥120 days treatment gap, without switching).

Additional data retrieved from the database included socio-demographic factors (age, sex, residential area, socioeconomic status {SES}, and smoking status) and baseline comorbidities according to MHS registries (cardiovascular disease, diabetes, hypertension, obesity, and osteoporosis). Depression and anxiety were defined according to antidepressants and benzodiazepines dispensed 180 days before the index date. The Charlson’s comorbidity index (CCI) was calculated at baseline. Additional data at baseline included years since AS diagnosis, visits to a primary care physician 180 days before the index date, and being hospitalized at least once 180 days before the index date.

### 2.4. Statistical Analyses

Patients’ disposition, baseline characteristics, adherence rates, discontinuation rates, type, and time to discontinuation are presented using descriptive statistics (*n*, %, mean ± SD or median, IQR, as appropriate). Adherence was assessed by treatment status and individual drug. Differences in adherence rates were assessed using Chi-square tests.

Discontinuation rate, type, and time to discontinuation in the first 12 months were assessed among patients with ≥12 months follow-up and presented by treatment experience status and individual drug. Time to treatment discontinuation by treatment experience status was also analyzed and plotted using Kaplan–Meier estimates. The log-rank test was used to evaluate statistical significance differences between individual drugs.

The risk of treatment discontinuation of each drug was estimated separately for the biologic-naïve and the biologic-experienced groups using a multivariable Cox proportional hazards regression model. The model was adjusted for age, sex, SES, time since diagnosis, and CCI.

Two-sided *p* < 0.05 was considered statistically significant. All statistical analyses were performed with IBM-SPSS V.25.0 standard statistical software for Windows and R V.3.5.

## 3. Results

Between January 2015 and December 2017, 343 eligible AS patients were identified (Table 1). The mean age was 44.6 years (SD ± 13.4) with a slight male predominance (57.7%) and for 257 patients (74.9%) the time since diagnosis at baseline was under two years. The most prevalent comorbidities were obesity (23.6%), depression or anxiety (22.7%), and hypertension (21.0%). Most patients (69.7%) were biologic-naive, and 482 lines of therapy were used during follow-up. Because only one patient used Tofacitinib, it was excluded from all analyses. Therefore, a total of 481 lines of treatment were included.

### 3.1. Patients’ Disposition

**Figure 1:**
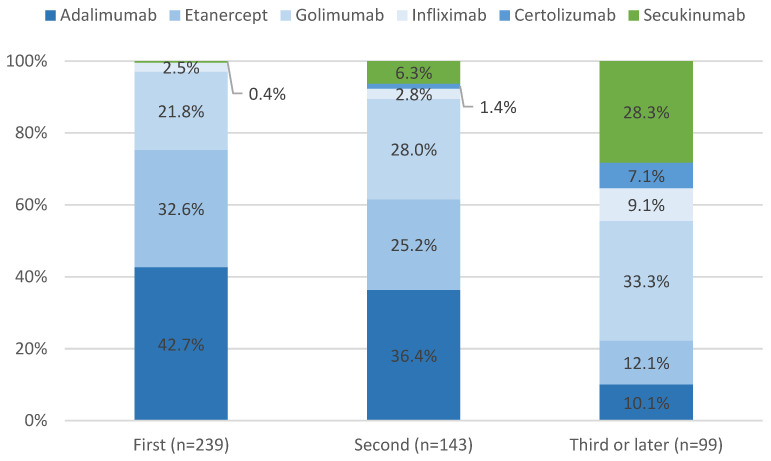
Patient disposition by treatment lines.

Among the 239 patients using their first line of therapy, the most common drug used was ADA (42.7%), followed by ETN (32.6%) and GOL (21.8%). The remaining 2.9% were treated with IFX (*n* = 6) and SEC (*n* = 1). Among the 143 patients on their second line of therapy (including biologic-naive patients who switched during follow-up), ADA (36.4%), ETN (25.2%), and GOL (28.0%) remained the most prevalent drugs. Figure 1 depicts the disposition of patients according to their treatment episode. The proportion of patients utilizing CTZ and IFX increased to 10.5%. Among the patients utilizing a third line of therapy (*n* = 99), we noticed a decrease in the proportion of patients prescribed ADA, ETN, and GOL (10.1%, 12.1%, and 33.3%, respectively), while the proportion of patients prescribed with SEC increased to 28.3%, along with an increment in the proportion of patients using IFX (9.1%) and CTZ (7.1%).

### 3.2. Adherence to Treatment

Among biologic-naïve patients (Table 2), the proportion of highly adherent patients (PDC ≥ 0.8) was similar (*p* > 0.9) and highest for ADA (71.6%), followed by ETN (69.2%) and GOL (63.5%). Among the biologic-experienced group, the highest adherence was recorded for SEC (75.7%), followed by ADA (69.4%), GOL (60.3%), and ETN (50.0%) (*p* < 0.001).

### 3.3. Discontinuation and Drug Survival

Table 3 displays the discontinuation rate in the first 12 months by biologic-experience status. Among the biologic-naïve group, the discontinuation rate was highest for GOL (34.6%), followed by ADA (30.4%) and ETN (28.2%) (*p* = 0.7). Among the biologic-experienced group, the discontinuation rate was highest for ETN (47.4%) and lowest for SEC (30.8%) (*p* = 0.3).

**Figure 2:**
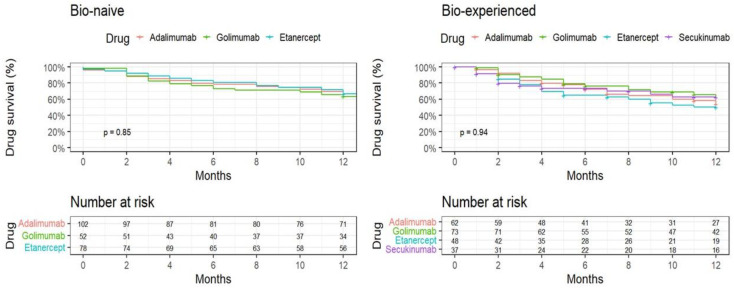
One year Kaplan–Meier plot time to discontinuation, by treatment experience status and individual drug.

The Kaplan–Meier analysis (Figure 2) shows similar survival rates for ADA, GOL, and ETN among the biologic-naïve group (*p* = 0.85). Among the biologic-experienced group, after 12 months, GOL and SEC displayed similar drug survival rates, followed by ADA and ETN; however, the results did not reach statistical significance (*p* = 0.94).

The multivariable models (Table 4) showed that in the biologic-naïve group, females had an increased risk for treatment discontinuation (HR 2.03, 95% CI 1.26–3.28). A similar risk for discontinuation was observed for GOL and ETN compared to ADA (HR 1.23, 95% CI 0.68–2.23; HR 0.89, 95% CI 0.51–1.55, respectively). Among the treatment-experienced group, longer disease duration was associated with reduced risk for discontinuation (HR 0.90, 95% CI 0.82–1.00). In the biologic-naïve group, GOL, ETN, and SEC had similar risk for discontinuation compared with ADA (HR 0.99, 95% CI 0.53–1.84; HR 1.38, 95% CI 0.73–2.61; HR 1.09, 95% CI 0.67–1.05, respectively).

## 4. Discussion

Our study aimed to evaluate adherence, drug survival, and risk for discontinuation of biologic-naïve and -experienced AS patients in a real-world setting.

Adherence to biologics in the first 12 months in the biologic-naïve group was similar (*p* = 0.497) across all medications of interest, with 63.5–71.6% of the patients being highly adherent. In the biologic-experienced group, the highest rate of highly adherent patients was recorded for SEC (75.7%) and the lowest for ETN (50.0%), reflecting significant differences between the different drugs (*p* < 0.001).

The relatively high adherence rate in both treatment groups is worth noting. We previously reported high adherence rates to ADA among patients with AS compared to other inflammatory diseases [24], postulating that the relative paucity at the time of biologics for AS (compared to rheumatoid arthritis or psoriatic arthritis) is a plausible explanation.

Interestingly, we have found that the highest adherence, together with the lowest discontinuation rate (30.8%), was among patients prescribed with SEC in comparison to TNF-αis. In addition, the proportion of patients treated with SEC increased gradually between lines of therapy, reaching 28% when prescribed as a third line of therapy or above. Taken together, we believe these properties reinforce the aforementioned tendency to maintain treatment when other options are becoming less available. At the same time, drug survival is an important proxy measure for the effectiveness of treatments for inflammatory diseases [15], and the adherence rate may also reflect the drug’s efficacy [25]. Therefore, the high proportion of highly adherent patients treated with SEC in our study and its relatively high level of drug survival in our study and in other studies [26,27] might indicate that SEC can be an effective treatment option for patients with AS.

As seen in previous studies [16,28], drug survival decreased with time. In our study, none of the drugs utilized by either biologic-naïve or biologic-experienced patients showed superiority in terms of drug survival or discontinuation risk.

Real-world data relating to SEC drug properties in patients with AS are currently scarce; however, our study sheds some light on the topic, showing similar drug survival between SEC and TNF-αis. Our findings are in line with a meta-analysis by Yu et al. [29], which reported comparable drug survival for all biologics for the treatment of AS. Another study retrospectively exploring the drug survival of SEC, either as the first treatment line or following TNF-αi exposure, was conducted by Diaconu et al. [30]. The authors found that drug survival was 59.7% at 12 months and 31.3% at 24 months, without significant differences in the median drug survival between biologic-naïve versus biologic-experienced subgroups.

In our study, the drug survival was similar for TNF-αis and SEC when given to biologic-experienced patients. These results are supported by other studies, showing that patients switching from either one TNF-αi to another [30,31,32] or to SEC [33] displayed fair survival rates.

In contrast to our findings, former studies found poor drug survival for biologic-experienced compared to biologic-naïve patients. According to a single-center observational study by Gyulas et al. [28], the overall survival time of biologic-naïve AS patients prescribed with TNF-αis was better in comparisons with those on their second treatment line [62.88 months (95% CI: 56.67–69.09) and 39.29 months (95% CI: 31.29–47.03), *p* = 0.05, respectively], yet the authors state that switching between TNF-αis is still a good therapeutic option for biologic-experienced patients. A real-life multicenter study by Krajewski et al. [34] reported similar results showing that the drug survival of all TNF-αis was shorter for the second than the first TNF-αi (mean difference 6.3 months, *p* < 0.001).

According to our findings, female biologic-naïve patients displayed an increased risk for treatment discontinuation. However, gender seems to be an equivocal factor in treatment discontinuation. According to Aturi et al. [35], the lack of adherence to treatment was not associated with sex, while in another study, male sex was associated with a better retention rate [36].

There are some limitations in our study. Due to the retrospective design, which is based on administrative data, some data could not be retrieved, including disease activity, the presence of extra-articular symptoms, and concomitant conventional synthetic DMARDs, which influence drug retention [37]. Also, the reasons for drug discontinuation by each subject and whether it was related to loss of efficacy, adverse events, or other factors could not be retrieved from the data. The study did not include all bDMARDs and tsDMARDS currently available for patients with AS. In addition, we did not distinguish AS from non-radiographic axial spondylarthritis, which shows conflicting results regarding treatment adherence [38,39]. The current study also has several strengths. We were able to follow patients for a long period of time and include almost all bDMARDs available for AS, including the IL-17i SEC. We used real-world data without any exclusion or randomization. Additionally, we were able to assess adherence, drug survival, and the risk for treatment discontinuation in treatment-naïve and treatment-experienced patients.

In conclusion, drug adherence, survival, and risk for discontinuation were similar for both TNF-αis and the IL-17i SEC, regardless of biologic-experience status. Etanercept was the TNF-αi with the lowest rate of adherent patients and the highest rate of discontinuation among biologic-experienced patients. The drug survival rate among biologic-experienced patients was similar to that of biologic-naïve patients, indicating that patients who discontinued their first biologic may benefit from switching to another. As these features are indirect methods to appraise drug efficacy and safety [40] in real-world settings, we believe caregivers can integrate these results into treatment considerations.

## Figures and Tables

**Table 1:** Baseline characteristics of AS patients (*n* = 343).

Mean age (SD)		44.62 (13.36)
Gender, male		198 (57.7%)
Socioeconomic status	Low	67 (19.5%)
	Medium	190 (55.4%)
	High	86 (25.1%)
Smoking status	Never	276 (80.5%)
	Ever	66 (19.2%)
	Unknown	1 (0.3%)
Time since diagnosis, years	Mean (SD)	1.59 (2.85)
	Median (IQR)	0.08 (0.00, 1.96)
Disease duration categories	Under 2 years	257 (74.9%)
	2–6 years	49 (14.3%)
	At least 6 years	37 (10.8%)
Biologic-experience status, naïve		239 (69.7%)
Lines of therapy used during follow-up	One	236 (68.8%)
	Two	81 (23.6%)
	Three and above	26 (7.6%)
Charlson comorbidity index	Mean (SD)	0.81 (1.35)
	Median (IQR)	0.00 (0.00, 1.00)
Comorbidities	Cardiovascular disease	25 (7.3%)
	Diabetes	19 (5.5%)
	Hypertension	72 (21.0%)
	Obesity	81 (23.6%)
	Osteoporosis	38 (11.1%)
	Depression and/or anxiety	78 (22.7%)
Mean number of visits to the PCP (SD)		7.70 (4.95)
At least one hospitalization		28 (8.2%)

**Table 2:** Adherence in the first 12 months by biologic-experience status.

**Biologic-Naive**						
	**Adalimumab** **(*n* = 102)**	**Etanercept** **(*n* = 78)**		**Golimumab** **(*n* = 52)**		***p*** **-Value ^1^**
PDC < 0.4	13 (12.7%)	12 (15.4%)		9 (17.3%)		0.497
PDC 0.4 ≤ 0.8	16 (15.7%)	12 (15.4%)		10 (19.2%)		
PDC ≥ 0.8	73 (71.6%)	54 (69.2%)		33 (63.5%)		
**Biologic-experienced**						
	**Adalimumab** **(*n* = 62)**	**Etanercept** **(*n* = 48)**	**Golimumab** **(*n* = 73)**		**Secukinumab** **(*n* = 37)**	***p*** **-Value ^1^**
PDC < 0.4	7 (11.3%)	11 (22.9%)	10 (13.7%)		3 (8.1%)	<0.001
PDC 0.4 ≤ 0.8	12 (19.4%)	13 (27.1%)	19 (26.0%)		6 (16.2%)	
PDC ≥ 0.8	43 (69.4%)	24 (50.0%)	44 (60.3%)		28 (75.7%)	

^1^ Chi-square test.

**Table 3:** Discontinuation rate and type in the first 12 months among patients with ≥12 months follow-up, by biologic-experience status.

**Biologic-Naive**						
		**Adalimumab** **(*n* = 102)**	**Golimumab** **(*n* = 52)**	**Etanercept** **(*n* = 78)**	**Secukinumab**	***p*-Value ^1^**
Discontinuation rate		31 (30.4%)	18 (34.6%)	22 (28.2%)	NA	0.7
Discontinuation type	Switch	21 (20.6%)	8 (15.4%)	13 (16.7%)		0.5
	Stop	10 (9.8%)	10 (19.2%)	9 (11.5%)		
	No discontinuation	71 (69.6%)	34 (65.4%)	56 (71.8%)		
Time to discontinuation, months	Mean (SD)	9.8 (3.9)	9.4 (4.0)	10.0 (3.7)		0.7
	Median (IQR)	12.0 (9.2, 12.0)	12.0 (6.0, 12.0)	12.0 (9.5, 12.0)		
**Biologic-experienced**						
		**Adalimumab** **(*n* = 50)**	**Golimumab** **(*n* = 59)**	**Etanercept** **(*n* = 38)**	**Secukinumab** **(*n* = 26)**	***p*-Value ^1^**
Discontinuation rate		19 (38.0%)	17 (28.8%)	18 (47.4%)	8 (30.8%)	0.3
Discontinuation type	Switch	11 (22.0%)	8 (13.6%)	10 (26.3%)	3 (11.5%)	0.5
	Stop	8 (16.0%)	9 (15.3%)	8 (21.1%)	5 (19.2%)	
	No discontinuation	31 (62.0%)	42 (71.2%)	20 (52.6%)	18 (69.2%)	
Time to discontinuation, months	Mean (SD)	9.3 (4.0)	10.2 (3.4)	8.4 (4.5)	9.7 (4.0)	0.2
	Median (IQR)	12.0 (6.0, 12.0)	12.0 (10.0, 12.0)	12.0 (4.0, 12.0)	12.0 (9.2, 12.0)	

^1^ Fisher’s exact test; Kruskal–Wallis rank sum test.

**Table 4:** HRs and 95% CIs for treatment discontinuation in the first 12 months among AS patients with ≥12 months follow-up, by biologic-experience status.

		Biologic-Naive			Biologic-Experienced		
		HR ^1^	95% CI ^1^	*p*-Value	HR ^1^	95% CI ^1^	*p*-Value
Age		1	0.98, 1.02	0.9	1.02	1.00, 1.04	0.076
Sex	Male	—	—		—	—	
	Female	2.03	1.26, 3.28	0.004	1.53	0.93, 2.52	0.093
Socioeconomic status	Low	—	—		—	—	
	Medium	1.34	0.68, 2.66	0.4	0.78	0.40, 1.50	0.5
	High	1.49	0.68, 3.25	0.3	0.66	0.30, 1.43	0.3
Time since diagnosis		0.7	0.40, 1.24	0.2	0.9	0.82, 1.00	0.044
Drug	Adalimumab	—	—		—	—	
	Golimumab	1.23	0.68, 2.23	0.5	0.99	0.53, 1.84	>0.9
	Etanercept	0.89	0.51, 1.55	0.7	1.38	0.73, 2.61	0.3
	Secukinumab				1.09	0.49, 2.40	0.8
Charlson comorbidity index		1.05	0.85, 1.29	0.7	0.84	0.67, 1.05	0.12

^1^ HR = Hazard Ratio, CI = Confidence Interval.

## Data Availability

The datasets generated during and/or analyzed during the current study are not publicly available because the data that support the findings of this study originate from Maccabi Healthcare Services, and restrictions apply to the availability of these data. Due to restrictions, these data can be accessed only by request to the authors and/or Maccabi Healthcare Services.
